# Assessment of left atrial function provides incremental value: the left atrial volumetric/mechanical coupling index in patients with chronic kidney disease

**DOI:** 10.3389/fcvm.2024.1407531

**Published:** 2024-07-09

**Authors:** Liqin Ji, Xue Gao, Weiwei Xiao, Shaomei Yu

**Affiliations:** ^1^ Guizhou Medical University, Guiyang, Guizhou, China; ^2^ Department of Ultrasound, Affiliated Hospital of Guizhou Medical University, Guiyang, Guizhou, China

**Keywords:** left atrial volumetric/mechanical coupling index, left atrium, left atrial function, chronic kidney disease, heart failure

## Abstract

**Background:**

Heart failure is a common cause of adverse cardiovascular outcomes in patients with chronic kidney disease (CKD). Left atrial (LA) characteristics are thought to be involved in the development of heart failure. However, LA assessment is complex. Though a variety of parameters have been defined, there is no single parameter that best defines LA function. Pilot data indicate that left atrial volumetric/mechanical coupling index (LACI) may be useful, but data with CKD are lacking.

**Aim:**

The objective of this study was to define LACI in a cohort of patients with CKD and to assess its value in evaluating LA function and predicting heart failure.

**Methods:**

A cohort of patients with CKD was enrolled at our hospital between 2021 and 2023. Follow-up was performed for heart failure. LACI is a volumetric to mechanical coupling index, calculated as the ratio of the LA volume index to the tissue-Doppler myocardial velocity at atrial contraction. Spearman’s rank correlation or Pearson’s correlation was used to calculate the correlation between LACI and echocardiographic/hemodynamic variables. Receiver operating characteristic curve (ROC) analysis was utilised to derive the area under the curve (AUC) for LACI, LVGLS, LASr, LASct and LASI for the detection of heart failure. Kaplan-Meier survival curves were employed to compare clinical outcomes based on LACI thresholds. A multivariable logistic regression analysis was employed to assess the relationship between risk factors and elevated LACI. Cox proportional hazards regression was used to identify risk factors for heart failure.

**Results:**

LACI showed a positive correlation with NT-proBNP, CK-MB, LAVI, E/e’ and LASI (*r* = 0.504, 0.536, 0.856, 0.541 and 0.509, *p* < 0.001); and a negative correlation with LASr (*r* = −0.509, *p* < 0.001). On the ROC analysis for the determination of heart failure, the AUC of LACI was comparable to those of LVGLS (0.588 vs. 509, *p* = 0.464), LASr (0.588 vs. 0.448, *p* = 0.132), LASct (0.588 vs. 0.566, *p* = 0.971) and LASI (0.588 vs. 0.570, *p* = 0.874). The cardiovascular risk factors increased by LACI were age, BMI, diabetes, triglycerides, LA size, LASr, LASI, E/A, E/e’ and EF (*p* < 0.05). During a median follow-up of 16 months (range, 6–28 months), the event-free survival curves demonstrated a higher risk of heart failure in the group with LACI > 5.0 (log-rank test: *P* < 0.001). LACI > 5.0 was an independent predictor of heart failure [OR: 0.121, 95% CI (0.020–0.740), *p* = 0.022].

**Conclusion:**

LACI may prove to be a valuable tool for assessing LA function in patients with CKD, and could be integrated into the routine assessment of LA for the purpose of prognostic assessment and clinical decision-making in patients with CKD.

## 1 Introduction

Chronic kidney disease (CKD) is a significant global health issue. Individuals with CKD have up to a 30-fold increased risk of cardiovascular mortality compared to the general population and are more likely to die from cardiovascular disease than to progress to dialysis stages (1, 2). Heart failure is one of the most common complications and the major cause of death in patients with CKD (3–5). Studies have demonstrated that echocardiographic findings, including left ventricular hypertrophy (LVH), reduced ejection fraction (EF), and diastolic dysfunction, are predictive of future risk of adverse cardiovascular events and all-cause mortality (6). LA is often considered a passive bystander of these pathophysiologic alterations (7). Yet, heart failure is not wholly caused by impairment of LV structure and function (8). More recently, there is evidence that LA dysfunction in patients with chronic kidney disease (CKD) is independently modulated by renal function, which may be indicative of a CKD-associated atrial myopathy (9). Furthermore, in conditions associated with systemic inflammation, such as CKD, alterations in LA function independent of diastolic dysfunction have been described (10).

Recent studies have highlighted new methods for LA assessment, suggesting that LA characteristics might influence heart failure outcomes (11, 12). Conventionally, LA function is assessed by volumetric measures. Diastolic tissue-Doppler myocardial velocity at atrial contraction (TDI-a’) is an established measure of LA mechanics, highly correlated to complex measures of LA performance (13, 14). Therefore, when assessing LA function, it is important to couple LA volume index (LAVI) to LA mechanical activity (TDI-a’), known as the left atrial volume/mechanical coupling index (LACI), which has shown promising results in other clinical contexts including atrial fibrillation, heart failure, stroke and acute ischaemic heart disease (15–19). However, it remains unclear whether LACI provides incremental information in CKD. We gathered such a cohort and measured LACI for the first time in the context of CKD to evaluate its determinants, and potential value.

## 2 Methods

### 2.1 Study participants

A total of 213 patients with chronic kidney disease (CKD) were enrolled in a prospective study conducted at our hospital between 2021 and 2023. Therefore, the study was a retrospective observational analysis of a prospective cohort database. CKD was classified based on the underlying cause, the glomerular filtration rate (GFR) category (G1–G5), and the stage of the disease (20). The following exclusion criteria were applied: significant mitral valve diseases, atrial fibrillation (AF) or flutter, congenital heart disease, or a history of cardiac surgery or permanent pacemaker implantation, cardiomyopathy, pericardial disease, patients with previous heart failure, inadequate echocardiographic acoustic windows, pregnancy, and age under 18 years old. Significant mitral valve disease was defined as more than moderate mitral valve stenosis or regurgitation on transthoracic echocardiography.

### 2.2 Clinical and echocardiographic data

The patient history and clinical characteristics were extracted from the electronic medical records without any modification. The glomerular filtration rate (GFR) was estimated using the Cockcroft-Gault formula. Furthermore, all patients underwent echocardiography within 48 h of admission.

All echocardiographic measurements were guided by the American Society of Echocardiography recommendations. The LA anteroposterior diameter (LAd) was evaluated from the parasternal long-axis view. The LA volume index (LAVI) corrected for body surface area was derived using the heart model, with the patient’s height and weight entered as inputs. In patients with sinus rhythm, diastolic filling was assessed using early (E) and late (A) inflow velocities, the E/A ratio, e’ (mean of septal and lateral wall derivation) and a’ (mean of septal and lateral wall derivation) derived from tissue Doppler, and the E/e’ ratio. LACI is a volumetric to mechanical coupling index, calculated as the ratio left atrial volume index/tissue-Doppler myocardial velocity at atrial contraction.

Speckle echocardiography was used with *in vivo* quantitative analysis software to automatically track myocardial motion and generate the region of interest (ROI), measured the LV and LA speckle tracking parameters. LA phasic strains included reservoir phase (LASr), conduit phase (LAScd) and contractility (LASct). The LA stiffness index (LASI = E/e’/LASr) was also calculated. The region of interest was subsequently adjusted in order to obtain the global longitudinal strain of the left ventricle (LVGLS). Digital echocardiographic data containing at least 3 consecutive beats were acquired, transferred to a server for storage and interpreted by 2 independent cardiologists blinded to each other's reading.

### 2.3 Follow- up data

Participants were followed for a median of 16 months (range: 6–28 months) with the primary outcome being rehospitalization for heart failure, defined by the Framingham criteria (21). All patients were followed up either in outpatient clinics or by telephone conversation.

### 2.4 Statistical analysis

Data were analyzed using SPSS 27.0. The results are presented as mean ± SD for the normally distributed variables and as median (IQR) for skewed variables. Correlations between LACI and echocardiographic/hemodynamic variables were calculated using Spearman's rank or Pearson correlation. Receiver operating characteristic (ROC) curve analysis was used to obtain the area under the curve (AUC) for LACI, LVGLS, LASr, LASct, and LASI for heart failure detection, with comparisons made using the Delong method. Survival rates (±SE) were estimated using the Kaplan–Meier method and compared using the log-rank test. Determinants of increased LACI were assessed by logistic regression and selected based on pathophysiologic links to LA function. Cox proportional hazards models identified risk factors for heart failure. Variables with *p* < 0.05 in univariate analysis were included in multifactorial analysis, with significance set at *p* < 0.05.

## 3 Results

### 3.1 Baseline characteristics

Among the 213 CKD patients, 129 (60.6%) were male and 84 (39.4%) were female, with an average age of 57.4 ± 14.3 years. Median follow up was 16 months (interquartile range 13–21 months). Overall 23 patients (10.8%) reached the end point. Baseline clinical and laboratory characteristics of study subjects are shown in Table 1, and echocardiographic measures are shown in Table 2.

**Table 1:** Demographic and laboratory characteristics.

Age (years)	49 (36, 59)
Sex (male/female) *N*	129/84
Height (cm)	162 (158, 169)
Body weight (kg)	61.75 (52.75, 67.35)
BMI (kg/m^2^)	23.15 (20.98, 24.97)
Systolic blood pressure (mmHg)	144.41 ± 23.63
Diastolic blood pressure (mmHg)	88.99 ± 16.19
Dialysis or not (*n*)	90/123
Haemodialysis (*n*, %)	71 (33.3%)
Peritoneal dialysis (*n*, %)	19 (8.9%)
Hypertension (*n*, %)	157 (73.7%)
Diabetes (*n*, %)	40 (32.9%)
Coronary artery disease (*n*, %)	8 (3.8%)
Smoking history (*n*, %)	68 (31.9%)
Drinking history (*n*, %)	56 (26.3%)
Potassium (mmol/L)	4.43 ± 0.76
Sodium (mmol/L)	140.56 ± 3.73
Chlorine (mmol/L)	107.37 ± 70.41
Calcium (mmol/L)	2.11 ± 0.27
Magnesium (mmol/L)	0.90 (0.81, 1.04)
Inorganic phosphate (mmol/L)	1.58 ± 0.60
Urea (mmol/L)	16.56 (8.98, 23.34)
Creatinine (umol/L)	639.69 ± 485.86
uric acid (umol/L)	437.76 ± 143.08
Cystatin C (mg/L)	4.49 ± 2.24
GFR (ml/min/1.73 m^2^)	29.88 ± 12.95
Total protein (g/L)	64.48 ± 11.64
Plasma albumin (g/L)	50.84 ± 193.71
CK-MB (U/L)	12.86 (9.86, 17.47)
CTnT (ng/ml)	0.031 (0.017, 0.07)
NT-proBNP (pg/ml)	2,679 (503.85, 13,732.00)
LDH (U/L)	227.00 (178.00, 273.50)
Triglyceride (mmol/L)	1.72 (1.21, 42.51)
Total cholesterol (mmol/L)	4.45 ± 1.60
HDL-C (mmol/L)	1.20 ± 0.93
LCL-C (mmol/L)	2.59 ± 1.29

**Table 2:** Echocardiographic characteristics.

LAd (mm)	36.00 (32.00, 40.00)
LVDd (mm)	48.00 (44.00, 52.00)
LAVI (ml/m^2^)	36.00 (26.00, 47.50)
RWT (mm)	0.44 (0.39, 0.48)
LV mass (g/m^2^)	225.77 (189.14, 283.54)
LVMI (g/m²)	141.35 (113.23, 141.35)
EF (%)	61.00 (56.00, 67.00)
E (cm/s)	87.00 (66.40, 110.50)
A (cm/s)	88.10 (70.65, 102.00)
E/A	0.98 (0.75, 1.27)
e’ (cm/s)	6.47 (5.11, 8.22)
E/e’	12.85 (9.40, 17.94)
TDI-a’ (cm/s)	8.84 ± 1.99
LACI (LAVI/TDI-a’)	3.98 (3.09, 5.37)
LASI (E/e'/LASr)	0.41 (0.22, 0.80)
LASr (%)	33.00 ± 14.32
LAScd (%)	140.56 ± 3.73
LASct (%)	−14.62 ± 8.96
LVGLS (%)	−16.25(−20.00, −13.00)

### 3.2 Correlations among LACI, NT-proBNP, CK-MB and echocardiographic parameters

**Figure 1:**
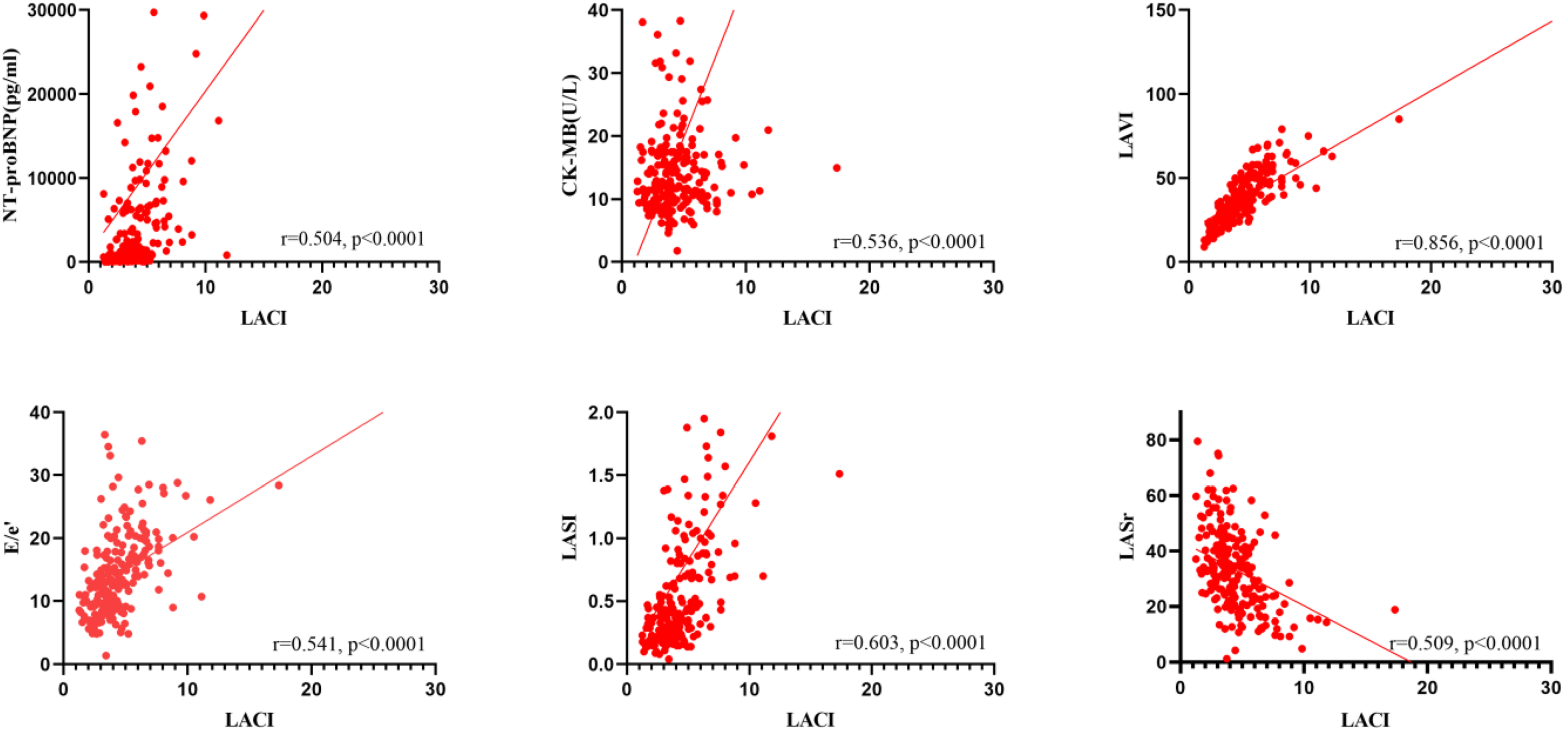
Correlations among LACI, NT-proBNP, CK-MB and echocardiographic parameters.

Figure 1 demonstrated that LACI was moderately correlated with NT-proBNP, CK-MB, E/e’, LASI, and LASr (*r* = 0.504, *r* = 0.536, *r* = 0.541, *r* = 0.603, *r* = −0.509, *P* < 0.0001) and highly correlated with LAVI (*r* = 0.856, *P* < 0.0001).

### 3.3 Prognostic performance of LACI

**Figure 2:**
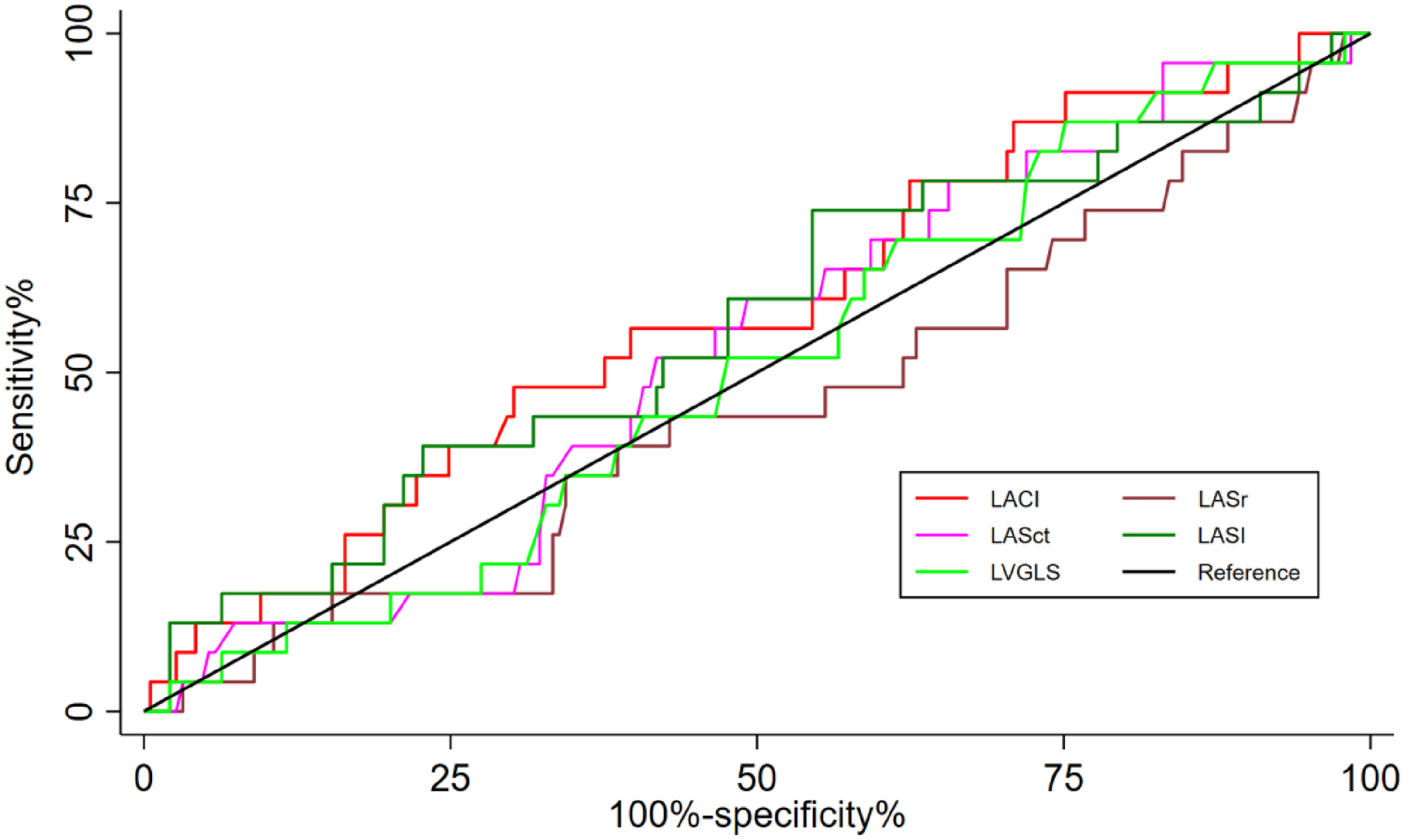
Differences in the prognostic impact of LACI, LVGLS, LASr, LASct, and LASI on hospitalisation for heart failure were compared between groups using the delong test at the 2-year time point, all *p* > 0.05.

Figure 2 showed the prognostic performance of LACI, LASr, LASct, LASI, and LVGLS for predicting heart failure during follow-up. The AUC of LACI for the entire study population was comparable to that of LVGLS (0.588 vs. 509, *P* = 0.464), LASr (0.588 vs. 0.448, *P* = 0.132), LASct (0.588 vs. 566, *P* = 0.971) and LASI (0.588 vs. 0.570, *P* = 0.874). A LACI of 5.0 was the best cut-off value to identify heart failure.

### 3.4 Determinants of increased LACI

Table 3 showed the increased cardiovascular risk factors for LACI. At multivariable analysis, LACI > 5 was significantly related to age [OR, 1.016 (95% CI, 1.021–1.102), *P* < 0.05], BMI[OR, 1.029 (95% CI, 1.004–1.055), *P* < 0.05], diabetes [OR, 1.319 (95% CI, 1.319–13.917), *P* < 0.05], triglycerides [OR, 0.425 (95% CI, 0.425–0.994), *P* < 0.05], LASr [OR, 0.877 (95% CI, 0.877–0.976), *P* < 0.05], LAd [OR, 1.16(95% CI, 1.16–1.479), *P* < 0.001], LASI [OR, 0.158 (95% CI, 0.158–0.939), *P* < 0.05], E/A [OR, 1.903 (95% CI, 1.903–25.808), *P* < 0.05], E/e’ [OR, 1.019 (95% CI, 1.019–1.24), *P* < 0.05].

**Table 3:** Multivariate logistic regression analysis was performed to analyze the determinants of LACI > 5.

Variables	B	Standard deviation	Wald	*P*	HR (95% CI)
Age (years)	0.059	0.019	9.258	0.002	1.061 (1.021–1.102)
Male (*n*, %)	−0.998	0.537	3.449	0.063	0.369 (0.129–1.057)
BMI	0.028	0.013	5.048	0.025	1.029 (1.004–1.055)
Hypertension history	−0.556	0.584	0.908	0.341	0.183 (0.183–1.800)
Diabetes history	1.455	0.601	5.859	0.015	1.319 (1.319–13.917)
Triglyceride (mmol/L)	−0.431	0.217	3.953	0.047	0.425 (0.425–0.994)
Cholesterol (mmol/L)	0.110	0.608	0.033	0.856	0.339 (0.339–3.678)
HDL-C (mmol /L)	0.073	0.728	0.010	0.920	0.258 (0.258–4.476)
HDL-C (mmol /L)	−0.137	0.259	0.280	0.596	0.525 (0.525–1.448)
LASr (%)	−0.078	0.027	8.134	0.004	0.877 (0.877–0.976)
LAd (mm)	0.270	0.062	18.884	0.000	1.160 (1.160–1.479)
LASI (E/e’/LASr)	−0.954	0.454	4.407	0.036	0.158 (0.158–0.939)
LVEF (%)	−0.035	0.030	1.329	0.001	0.910 (0.910–1.025)
E/A	1.947	0.665	8.571	0.003	1.903 (1.903–25.808)
E/e’	0.117	0.050	5.450	0.020	1.019 (1.019–1.240)
RWT	0.588	2.861	0.042	0.837	0.007 (0.007–490.280)
LVM (g/m^2^)	−0.006	0.004	2.128	0.145	0.986 (0.986–1.002)
LVMI	0.000	0.001	0.064	0.800	0.999 (0.999–1.002)

### 3.5 LACI > 5 as a predictor of clinical outcomes

**Figure 3:**
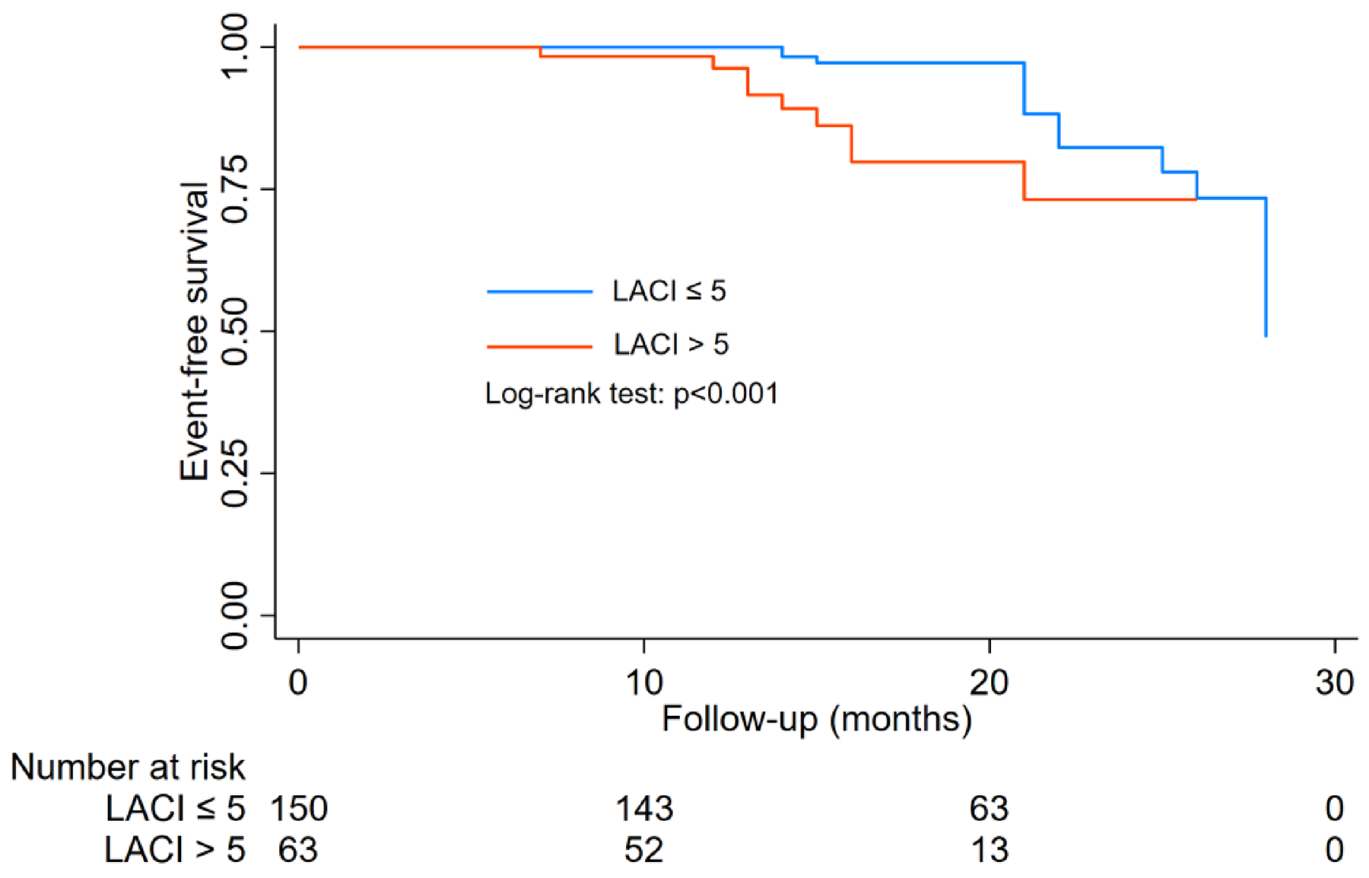
Kaplan–Meier and log-rank comparisons of heart failure according to LACI cut-off value.

During a median follow-up of 16 months (range, 6 to 28 months), the group with LACI > 5.0 had a higher incidence of heart failure than the group with LACI ≤ 5.0 (9.3% vs. 14.3%, *P* < 0.05). Event-free survival curves also showed a higher risk of heart failure in the group of LACI > 5.0 (log-rank test; *P* < 0.001, Figure 3). In Cox proportional hazards multivariate analysis, LACI > 5 was an independent predictor of heart failure (Table 4).

**Table 4:** Multivariate COX proportional hazards regression models predict heart failure during follow up.

Variable	Univariate analysis			Multivariate analysis		
	OR	95% CI	*P*	OR	95% CI	* P*
Age (years)	0.992	0.967–1.016	0.502			
Male (*n*)	0.47	0.184–1.200	0.114			
Hypertension history	0.631	0.251–1.588	0.328			
Diabetes history	1.319	0.516–3.374	0.563	0.254	0.065–0.985	**0**.**048**
GFR (ml/min/1.73 m^2^)	0.955	0.910–1.002	0.061			
Cystain C (mg/L)	1.236	0.998–1.530	0.052			
CTnT (ng/ml)	0.682	0.009–50.279	0.861			
NT-proBNP (pg/ml)	1.000	1.000–1.000	0.134			
Lad (mm)	1.080	0.989–1.179	0.086			
LAVI (ml/m^2^)	1.039	1.009–1.069	**0**.**01**			
E(cm/s)	1.007	0.8992–1.022	0.339			
A(cm/s)	1.001	0.988–1.103	0.911			
E/A	1.451	0.495–4.258	0.497			
TDI-a’ (cm/s)	0.956	0.784–1.165	0.656			
LVEF (%)	0.907	0.840–0.979	**0**.**013**			
E/e’	1.052	0.997–1.110	**0**.**044**			
LACI > 5	0.171	0.060–0.493	**0**.**001**	0.121	0.020–0.740	**0**.**022**
LASr (%)	0.969	0.933–1.007	0.104			
LAScd (%)	1.032	0.970–1.097	0.321			
LASct (%)	1.038	0.980–1.099	0.206			
LVGLS (%)	1.009	0.908–1.122	0.861			
LASI (E/e’/LASr)	1.496	0.931–2.404	0.096			

Variables included in the multivariate analysis included age, sex, hypertension history, diabetes history, and LAVI (ml/m^2^) LVEF (%), E/e’, and LACI > 5, which *p*-values less than 0.5 in the univariate analysis and are marked in bold.

## 4 Discussion

This study was the first to measure LACI in the context of CKD and to assess the determinants of LACI and the potential incremental value of LACI. The main findings were as follows: (1) LACI correlated well with NT-proBNP, CK-MB, and echocardiographic diastolic parameters; (2) Higher LACI corresponded to weaker atrial mechanical activity for larger volume index, with higher E/e’ and with several markers of increased volume/pressure overload. (3) LACI demonstrated comparable predictive ability to LVGLS, LASr, LASct and LASI in patients with CKD. (4) Risk factors for increased LACI included age, BMI, diabetes, triglycerides, left atrial size, LASr, LASI, EF, E/A, and E/e’. (5) LACI > 5 was an independent predictor of heart failure. Therefore, the atrial coupling index measured by LACI should be part of routine LA assessment in clinical practice, prognostic assessment, and clinical decision-making in patients diagnosed with CKD.

The LA has an important role in modulating LV performance, contributing up to a third of cardiac output (22). LA functions in a three-phase cycle, consisting of a reservoir, conduit, and booster pump, which dynamically interplay with the LV (23). In the early preclinical stages of heart failure, there is a compensatory increase in active LA contribution to LV filling, known as LA booster pump function (8, 24). This contributes to the maintenance of cardiovascular hemodynamics, cardiac output, and neurohumoral balance (25, 26). In patients with CKD, the LA is more sensitive to fluid overload and increased LV filling pressure, which can lead to a decrease in LA contribution to diastolic performance and an increase in LA volume due to elevated LV filling pressure (25, 27). Chronic exposure to elevated LV filling pressure can induce fibrotic changes in the LA, resulting in reduced compliance (28, 29). Previous researchers have found a significant correlation between impaired LV compliance and the degree of LA wall fibrosis as assessed by delayed enhancement magnetic resonance imaging (MRI) (30). Consistent with previous studies (31), our findings also revealed that patients with CKD have impaired LA reserve strain and increased LA stiffness index. The results of a study conducted by Daniel A. Morris et al. (32) demonstrated that the incorporation of LA strain into LAVI significantly enhanced the ability to identify patients at risk of LV diastolic dysfunction with preserved LVEF. This may also account for the similar predictive efficacy observed between LACI and both the LASI and LASct in this study. This is likely due to the impairment of the LA pump function and the concomitant decrease in compliance and increase in stiffness.

The volumetric analysis of the LA using LAVI is an adequate method to estimate the cumulative effect of increased LV filling pressures over time (33). Previous studies found that higher severity of CKD stages was associated with higher values of LA volume (34). The LAVI as an indicator of LA remodeling in chronic kidney disease (35). In addition, diastolic tissue-Doppler myocardial velocity at atrial contraction (TDI-a’) is an established measure of LA mechanics (36). A’ value measurement is a non-invasive and useful method for risk stratification in HFpEF as confirmed by Fumi Oike et al (37). Diastolic late mitral annular velocity (a’) measured by transthoracic echocardiography is reported to represent LA pump function and the severity of LA remodelling (38). It is highly correlated with complex measures of LA performance and can be measured in routine practice (39). Thus, when assessing LA function, it is crucial to combine a measurement of atrial stretch (LAVI) with a measurement of LA mechanical activity (TDI-a’, which is less affected by LV diastolic function than A wave). These complex interactions were consistent with our findings. Cardiovascular risk factors (age, BMI, diabetes mellitus, hyperlipidaemia) contribute to increased LACI, and increased LACI is also associated with reduced LA compliance (reduced LA strain and increased stiffness) and LV diastolic dysfunction. In addition, short-term follow-up results suggested a relationship between LACI and poor prognosis in heart failure.

It should be noted that the study is subject to several limitations. Firstly, the study was conducted at a single centre with a relatively small sample size, which may have implications for the validation of the methodology and the validity of the data. Secondly, due to our short follow-up period, many patients had not yet experienced an endpoint event. We hope that future prolonged follow-up may yield more significant differences and prognostic assessments. Furthermore, the low reproducibility of left atrial volume measurements reported in some of the literature should not be overlooked (40). Finally, patients with advanced chronic kidney disease are more likely to have severe hypertension. Consequently, the efficacy of treatment for hypertension may have influenced the results of our study.

## 5 Conclusions

LACI is a valuable tool for assessing left atrial function in CKD patients, offering incremental prognostic value beyond traditional measures. It should be included in routine evaluations, with further research needed to standardize clinical thresholds.

## Data Availability

The original contributions presented in the study are included in the article/Supplementary Material, further inquiries can be directed to the corresponding author.
